# Spectrochemical analysis of blood combined with chemometric techniques for detecting osteosarcopenia

**DOI:** 10.1038/s41598-023-36834-6

**Published:** 2023-06-15

**Authors:** Tales Gomes da Silva, Camilo L. M. Morais, Marfran C. D. Santos, Leomir A. S. de Lima, Raysa Vanessa de Medeiros Freitas, Ricardo Oliveira Guerra, Kássio M. G. Lima

**Affiliations:** 1 Institute of Chemistry, Biological Chemistry and Chemometrics, Federal University of Rio Grande do Norte, Natal, 59075-970 Brazil; 2 Federal Institute of Education, Science and Technology of Sertão Pernambucano, Floresta, 56400-000 Brazil; 3 Estácio de Sá Goiás, North Regional, Goiânia, GO 74063-010 Brazil; 4 Postgraduation Program in Health Sciences, Federal University of Rio Grande do Norte, Natal, 59075-970 Brazil; 5 Postgraduation Program in Physiotherapy, Federal University of Rio Grande do Norte, Natal, 59075-970 Brazil; 6 Department of Physiotherapy, Federal University of Rio Grande do Norte, Natal, 59075-970 Brazil

**Keywords:** Bioinformatics, Optical spectroscopy, Health care

## Abstract

Among several complications related to physiotherapy, osteosarcopenia is one of the most frequent in elderly patients. This condition is limiting and quite harmful to the patient’s health by disabling several basic musculoskeletal activities. Currently, the test to identify this health condition is complex. In this study, we use mid-infrared spectroscopy combined with chemometric techniques to identify osteosarcopenia based on blood serum samples. The purpose of this study was to evaluate the mid-infrared spectroscopy power to detect osteosarcopenia in community-dwelling older women (*n* = 62, 30 from patients with osteosarcopenia and 32 healthy controls). Feature reduction and selection techniques were employed in conjunction with discriminant analysis, where a principal component analysis with support vector machines (PCA–SVM) model achieved 89% accuracy to distinguish the samples from patients with osteosarcopenia. This study shows the potential of using infrared spectroscopy of blood samples to identify osteosarcopenia in a simple, fast and objective way.

## Introduction

Osteosarcopenia is defined by the *European Working Group on Sarcopenia in Older People* (EWGSOP) as a progressive and generalized musculoskeletal disorder that is related to physical disability, falls, fractures, and death^1^. Osteosarcopenia is a clinical condition often present in people at domestic risk, being considered a factor for several independent health problems, such as difficulties in performing basic and instrumental activities for daily living. In addition, regardless from the age, patients with osteosarcopenia have significantly more expenses in cases of hospitalization, taking up to 5 times more costs than those who do not have this condition^2^.

A review carried out in 2018 by the consensus proposed by the EWGSOP claim the reduction of muscle strength, called dynapenia, as the primary parameter of osteosarcopenia, having its diagnostics confirmed by the presence of reduced muscle mass (muscle amount) and/or by the reduction of physical performance (muscular quality). The prevalence of osteosarcopenia according to these criteria shows wide variety due to differences in the studied population and due to different methods employed to evaluate the diagnosis criteria^3^. The reference techniques employed for osteosarcopenia diagnosis are Magnetic Resonance Imaging (MRI) and Computed Tomography (CT) scans. However, both techniques are expensive, cause much discomfort to patients, and often are only employed in late-diagnosis. Therefore, new approaches to simplify the diagnosis and allow early-detection of osteosarcopenia are much welcome.

New analytical approaches employing biospectroscopy have played an important role in clinical diagnosis^4,5^. These approaches make use of vibrational spectroscopy techniques to analyse biological materials, since most molecules formed by covalent bonds absorb infrared (IR) radiation. Among these molecules, there are organic compounds containing important features of biological interest. Attenuated total reflection Fourier transform infrared (ATR-FTIR) spectroscopy allows a fast and non-destructive analysis of tissues, cells or biofluids^4,6^. For biofluids, a very small volume of sample is required for analysis, where microliters of sample can be used for measurement^4^. FTIR spectroscopy has been used to diagnose different types of cancer^7^, viruses^8^ and other conditions^9^.

Chemometric techniques have been widely used as a way for analysing spectroscopy data. Feature selection and classification methods have been used to analyse biological datasets with high data complexity due to the large amount of information acquired through the equipment. Some of the algorithms employed to reduce these data are the principal component analysis (PCA) and the successive projections algorithm (SPA). PCA is an unsupervised analysis algorithm capable of reducing the original and high-dimensional data into a small number of principal components (PCs), where each PC represents a part of the original data variance; while the SPA deterministically selects the variables that best differentiate the groups through the reduction of the data multicolinearity^10^.

Multivariate classification techniques can be applied to distinguishing the samples based on their spectrochemical profiles, even in the presence of unknown sources of variation or subtle spectral differences between the samples. Among the supervised classification techniques, linear discriminant analysis (LDA), quadratic discriminant analysis (QDA), and support vector machines (SVM) are widely employed since these can discriminate highly-complex data with a low risk of overfitting^10^. These algorithms are used here to differentiate between control and osteosarcopenia samples using the spectroscopic data collected for both groups in a case–control classification study.

## Materials and methods

### Samples

Blood samples (*n* = 62, being 32 healthy controls and 30 from patients with osteosarcopenia) were obtained from patients with informed consent. The patients were diagnosed based on Dual-energy X-ray Absorptiometry (DXA), which is a method recommended by the EWGSO^1^.The study was approved by the Research Ethics Committee of the Federal University of Rio Grande do Norte (UFRN) under number 2.368.206 following international and national standards (Resolution 466/12 of the National Council of Health) for research with human beings. Each elderly woman invited to participate in the research was informed about the objective and procedures to be adopted and were invited to sign the Informed Consent Form. The interviewers read the Informed Consent Form to the elderly and clarified any doubts about all stages of the process. All the patients in this study met the following eligibility criteria—inclusion criteria: (1) ability to walk alone for at least 400 m with or without auxiliary equipment, (2) absence of cognitive impairment (evaluated by the Leganés cognitive test with cut-off score above 22), (3) no history of cancer in the last 5 years, and, (4) no acute inflammatory or immunological condition, such as rheumatoid arthritis or systemic lupus erythematosus. The exclusion criteria were: (1) orthopedic or neurological deficiencies that could interfere with test results, (2) lack of regular physical activity (less than 3 times a week), and, (3) use of immunosuppressive drugs and/or corticoids in the last 3 months. The patients were filtered by these eligibility criteria before sample collection, so only patients suitable for the study were considered. The collected blood samples were centrifuged at 4000 rpm for 10 min to obtain the blood serum, which was kept in storage aliquots at − 80 ºC for further analysis. Before spectrometric analysis, all samples were thawed at room temperature for 30–40 min and then the protein precipitation process was performed. Precipitation was performed by adding 1.5 µL of 7 M perchloric acid to a 100 µL aliquot of serum. The aliquot was vortexed (FlexVortex 2, Loccus®) for 15 s, and centrifuged for 12 min at 12,000 rpm at 4 °C. The supernatant was then used for analysis (1 drop, approx. 50 μL). Although most of the proteins were precipitated, the sample may still contain proteins residues and small proteins, such as myokines which are fundamental for osteosarcopenia pathogenesis^11^.

### ATR-FTIR spectroscopy

The spectral acquisition was performed using a FTIR IRAffinity-1 spectrometer (Shimadzu Corporation, Japan) coupled to an ATR module containing a diamond crystal as the reflectance element. Measurements were made with 32 co-addition scans and 4 cm^−1^ spectral resolution. The spectral data were acquired in the 4000–600 cm^−1^ wavenumber range. The samples (10 µL) were applied directly on top of the ATR crystal for measurement. At the beginning of the experiment, the ATR crystal was cleaned with a mixture of ethanol 70% v/v and acetone p.a. (1:1); and, before each new sample, the crystal was cleaned with ethanol 70% v/v only. A new background spectrum was acquired before each new sample. Samples were measured in triplicate.

### Multivariate analysis

The spectral data were entire processed in the MATLAB 2014b environment (MathWorks, Inc., USA) using the PLS Toolbox version 7.9.3 (Eigenvector Research, Inc., USA) and lab-made routines. Firstly, the samples were dived into training (70%) and test (30%) sets using the Kennard-Stone (KS) algorithm^12^. The training samples were used for model construction and cross-validation, while the testing samples for final model evaluation. The spectral data were pre-processed by Savitzky-Golay smoothing (window of 5 points, 2nd order polynomial fitting) and automatic-weighted least squares baseline correction. Other pre-processing, including normalization procedures, were also tested but resulted in lower accuracies. The best pre-processing is presented herein. The replica pre-processed spectra were averaged for each sample, so the analysis was performed on a sample-basis. The data were also mean-centered before analysis.

The following classification algorithms based on PCA and SPA were used to analyse the pre-processed spectral data: PCA-LDA (principal component analysis with linear discriminant analysis), PCA-QDA (principal component analysis with quadratic discriminant analysis), PCA-SVM (principal component analysis with support vector machines), SPA-LDA (successive projections algorithm with linear discriminant analysis), SPA-QDA (successive projections algorithm with quadratic discriminant analysis), and SPA-SVM (successive projections algorithm with support vector machines).

PCA is one of the best well-known methods of reducing variables for large volumes of data, where a large number of spectral variables are reduced to a few number of PCs, containing scores and loadings^13^.The scores reflect the variance found with regard to the samples, while the loadings show the most important variables related to the scores construction. The scores and loading matrices are obtained after the decomposition performed by PCA on the pre-processed spectral matrix as follows:

1 $$\mathbf{X}=\mathbf{T}{\mathbf{P}}^{\mathrm{T}}+\mathbf{E}$$

where $\mathbf{T}$ represents the scores matrix; $\mathbf{P}$ represents the loading matrix; and $\mathbf{E}$ represents the residual matrix for total reconstruction of the pre-processed spectral matrix $\mathbf{X}$. As the scores represent the samples in the PC space, they can be used as input data for classification algorithms as in LDA, QDA and SVM.

SPA, on the other hand, performs a discrete selection of variables, selecting the variables that best differentiate the groups through the inverse of a cost function $\mathrm{G}$, represented below^14^:

2 $$\mathrm{G}=\frac{1}{\mathrm{Nv}} \sum_{\mathrm{n}=1}^{\mathrm{Nv}}{\mathrm{g}}_{\mathrm{n}}$$

where $\mathrm{Nv}$ is the number of validation samples and ${\mathrm{g}}_{\mathrm{n}}$ is defined as follows:

3 $${\mathrm{g}}_{\mathrm{n}}=\frac{{\mathrm{r}}^{2}({\mathrm{x}}_{\mathrm{n}},{\mathrm{m}}_{\mathrm{I}(\mathrm{n})} )}{{\mathrm{m}}_{\mathrm{I}(\mathrm{m})\ne \mathrm{I}(\mathrm{n})}{\mathrm{r}}^{2}({\mathrm{X}}_{\mathrm{n}},{\mathrm{m}}_{\mathrm{I}(\mathrm{m})})}$$

The numerator of Eq. 3 is the squared Mahalanobis distance between the sample *n*, ${\mathrm{x}}_{\mathrm{n}}$, and the center of the true class (${\mathrm{m}}_{\mathrm{I}(\mathrm{n})}$); and, the denominator represents the squared Mahalanobis distance between the sample ${\mathrm{x}}_{\mathrm{n}}$ and the center of the closest wrong class (${\mathrm{m}}_{\mathrm{I}(\mathrm{m})}$).

LDA and QDA are algorithms based on the Mahalanobis distance calculation between the samples. As the main difference between them, in LDA, it is assumed that all classes have well-defined and similar variance structures. In QDA, it is assumed that the classes do not have similar variance structures, thus, the covariance matrix is calculated individually for each analysed class^15^. The LDA (${\mathrm{L}}_{\mathrm{ik}}$) and QDA (${\mathrm{Q}}_{\mathrm{ik}}$) classification scores can be defined in a non-Bayesian form by the following Eqs.^16^:

4 $${\mathrm{L}}_{\mathrm{ik}}={\left({\mathbf{x}}_{\mathrm{i}}- {\overline{\mathbf{x}} }_{\mathrm{k}}\right)}^{\mathrm{T}}{\mathbf{C}}_{\mathrm{pooled}}^{-1}({\mathbf{x}}_{\mathrm{i}}- {\overline{\mathbf{x}} }_{\mathrm{k}})$$

5 $${\mathrm{Q}}_{\mathrm{ik}}={\left({\mathbf{x}}_{\mathrm{i}}-{\overline{\mathbf{x}} }_{\mathrm{k}}\right)}^{\mathrm{T}}{\mathrm{C}}_{\mathrm{k}}^{-1}({\mathbf{x}}_{\mathrm{i}}-{\overline{\mathbf{x}} }_{\mathrm{k}})$$

where ${\mathbf{x}}_{\mathrm{i}}$ is the response vector for a given *i*-th sample; ${\overline{\mathbf{x}} }_{\mathrm{k}}$ is the mean response vector for the *k*-th class; ${\mathbf{C}}_{\mathrm{pooled}}$ is the pooled covariance matrix; and ${\mathbf{C}}_{\mathrm{k}}$ is the calculated variance matrix for the *k*-th analysed class.

SVM is a supervised classification algorithm which transforms the original data into a new feature space using a kernel function that maximises, often non-linearly, the boundaries between the samples in their respective groups^17^. Among the main kernel functions, we have the radial basis function (RBF). The RBF function is calculated as follows^18^:

6 $$K\left({\mathbf{x}}_{\mathbf{i}},{\mathbf{z}}_{j}\right)=\mathrm{exp}(-\gamma \Vert {\mathbf{x}}_{i}-{\mathbf{z}}_{j}^{2}\Vert )$$

where ${\mathbf{x}}_{\mathbf{i}}$ and ${\mathbf{z}}_{j}$ are sample observations and $\gamma$ is the parameter that determines the RBF width.

The SVM classification was performed using the best training parameters obtained from cross-validation (venetian blinds with 10 data splits). The SVM classification takes the form:

7 $$f\left(\mathrm{x}\right)=\mathrm{sign}\left(\sum_{\mathrm{i}=1}^{{N}_{\mathrm{SV}}}{\alpha }_{\mathrm{i}}{y}_{\mathrm{i}}K\left({\mathbf{x}}_{\mathbf{i}},{\mathbf{z}}_{j}\right)+b\right)$$

where ${N}_{\mathrm{SV}}$ is the number of support vectors, ${\alpha }_{\mathrm{i}}$ is the Lagrange multiplier, ${y}_{\mathrm{i}}$ is the training class membership (± 1), $K\left({\mathbf{x}}_{\mathbf{i}},{\mathbf{z}}_{j}\right)$ is the kernel function, and $b$ is the bias parameter.

### Model validation

The models were validated based on quality parameters calculated for the test samples. The accuracy, sensitivity, specificity, F-Score and G-Score were calculated as follows^19^ :

8 $$\text{Accuracy }\left(\mathrm{AC}\right)=\left(\frac{\mathrm{TP}+\mathrm{TN}}{\mathrm{TP}+\mathrm{FP}+\mathrm{TN}+\mathrm{FN}}\right)\times 100$$

9 $$\text{Sensitivity }\left(\mathrm{SENS}\right)=\left(\frac{\mathrm{TP}}{\mathrm{TP}+\mathrm{FN}}\right)\times 100$$

10 $$\text{Specificity }\left(\mathrm{SPEC}\right)=\left(\frac{\mathrm{TN}}{\mathrm{TN}+\mathrm{FP}}\right)\times 100$$

11 $$\text{F-Score }\left(\mathrm{FS}\right)=\left(\frac{2\times \mathrm{SENS}\times \mathrm{SPEC}}{\mathrm{SENS}+\mathrm{SPEC}}\right)$$

12 $$\text{G-Score }\left(\mathrm{GS}\right)= \sqrt{\mathrm{SENS}\times \mathrm{SPEC}}$$

where FP stands for false positive, FN for false negative, TP stands for true positive, and TN for true negative.

## Results

**Figure 1:**
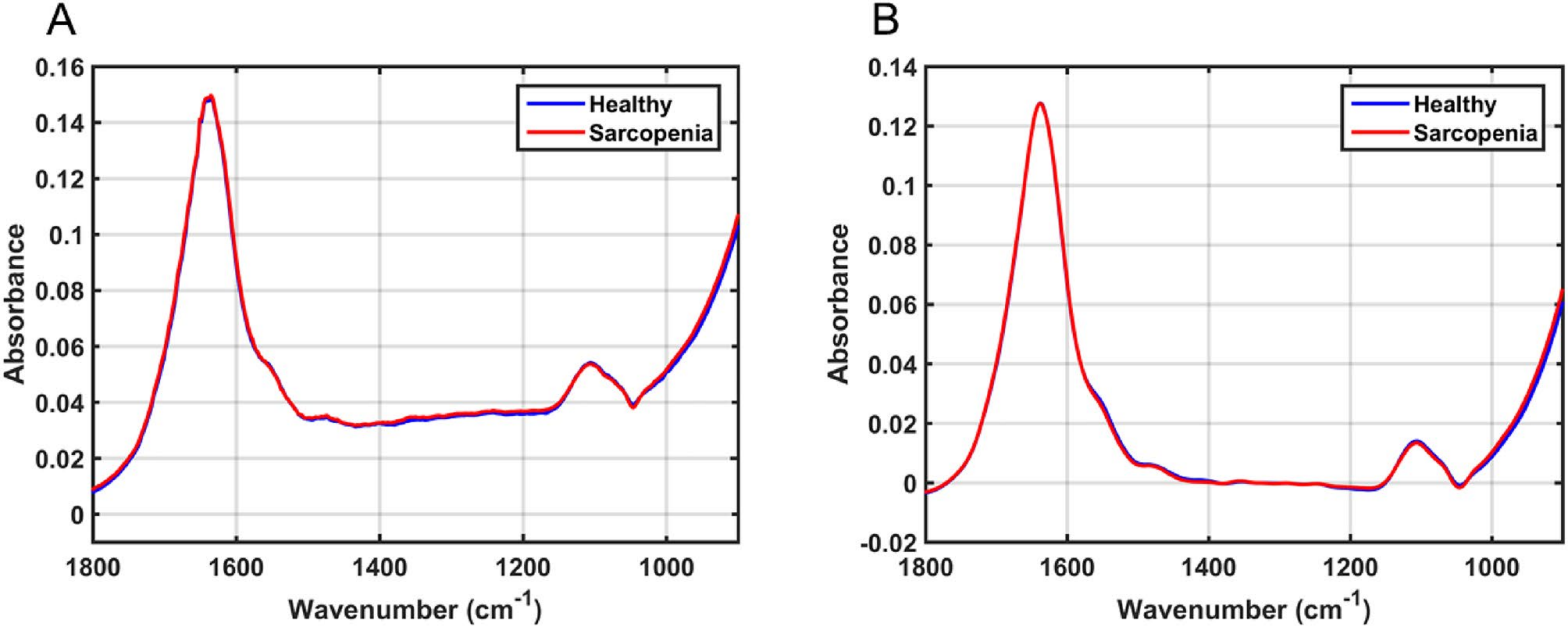
(**A**) Average raw spectra for healthy controls and osteosarcopenia samples; and, (**B**) averaged pre-processed spectra (Savitzky-Golay smoothing and baseline correction) for healthy controls and osteosarcopenia samples in the biofingerprint region (1800–900 cm^−1^).

In this study, 62 samples were analysed, including 32 healthy controls and 30 samples from patients with osteosarcopenia. The ATR-FTIR technique was used to obtain spectra from the blood serum of these patients. The spectra were analysed in the biofingerprint region (1800–900 cm^−1^), in which there are many absorption bands related to important biomolecules. For example, the amide I peak (~ 1650 cm^−1^) related to proteins^4^. The raw and pre-processed average spectra for the dataset are shown in Fig. 1A and B, respectively.

The spectral data were pre-processed by Savitzky-Golay smoothing and baseline correction, followed by mean-centering. The pre-processed spectral data were subjected to chemometric analysis by various classification techniques (PCA-LDA, PCA-QDA, PCA-SVM, SPA-LDA, SPA-QDA, SPA-SVM). Data processing is applied as a strategy to extract important spectral information to differentiate healthy controls from osteosarcopenia samples.

**Figure 2:**
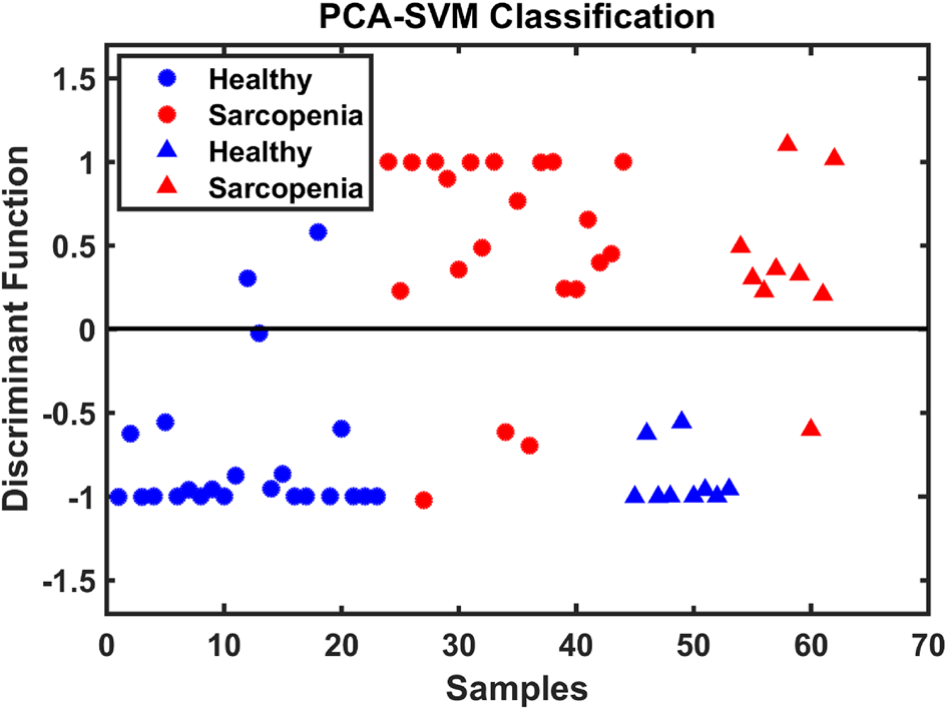
Discriminant function for PCA-SVM showing training (o) and testing (∆) samples.

For model construction, the pre-processed spectral data were divided into sets where 70% of the samples were used for training and 30% for testing using the KS uniform sample selection algorithm. Figures of merit (accuracy, sensitivity, specificity, F-Score and G-Score) were calculated to evaluate the performance of the model in relation to the prediction of samples used in the test set. Accuracy represents the total number of samples correctly classified considering true and false negatives; sensitivity represents the proportion of correctly classified positives; and specificity represents the proportion of negatives that are correctly classified. The statistical results calculated for the prediction set is shown, with the best model in bold, in Table 1. The best results after evaluating the test samples were obtained using the PCA-SVM model. The discriminant function that demonstrates the classes’ separation can be seen in Fig. 2.

**Table 1:** Figures of merits (FOM) for the tested models, where AC stands for accuracy, SENS for sensitviity and SPEC for specificity. The best model is highlighted in bold.

FOM	PCA			SPA		
	LDA	QDA	SVM	LDA	QDA	SVM
AC	56	72	**89**	33	61	72
SENS	56	67	**89**	22	33	44
SPEC	56	78	**89**	44	89	100
F-SCORE	56	72	**89**	30	48	62
G-SCORE	56	72	**89**	31	62	67

In terms of classification, the PCA-based models were built with a single PC capturing a variance of approximately 43% of the dataset. When only one score component was calculated for each sample with its pre-processed spectrum, it was possible to obtain, through the SVM classification algorithm, 89% for all figures of merit analysed, which is a relevant and important value for the distinction between the groups.

**Figure 3:**
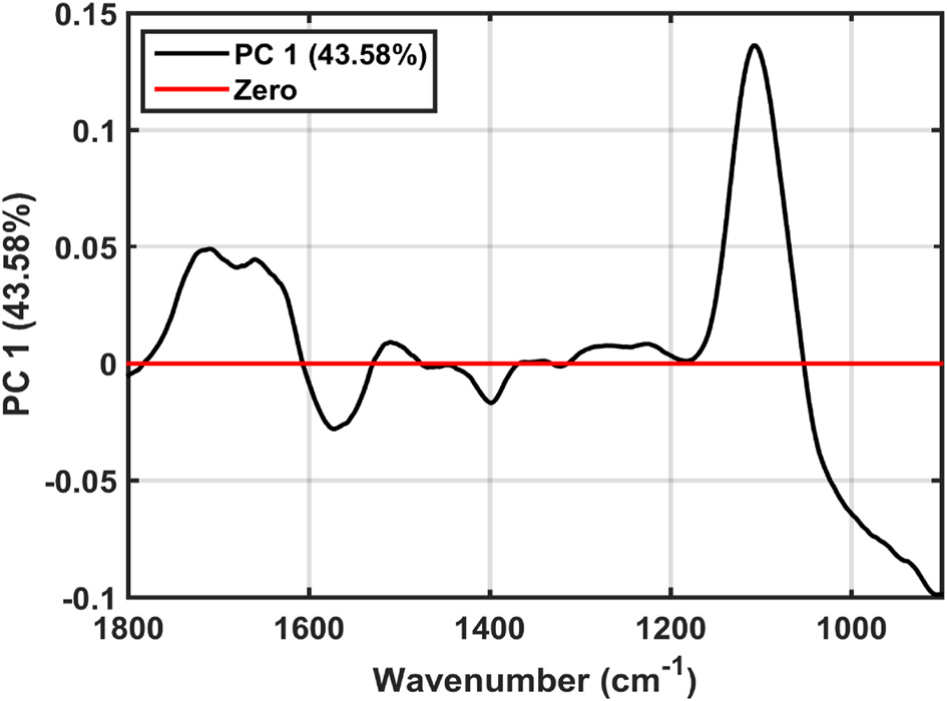
PCA loadings on PC1. The percentage inside parenthesis show the explained variance.

The loadings on the first PC, from the PCA-SVM model, were used to identify the most important variables for differentiation of the classes. The loadings peaks, selected as the region of greatest importance, were identified in the wavenumber regions of 1711, 1661, 1574, 1510, 1398, 1273, 1225, 1107, and 906.5 cm^−1^. The loadings graph is shown in Fig. 3. The attempt to assimilate these variables was carried out based on the study by Movasaghi et al*.*^20^ and is summarized in Table 2. Here, the assignments were performed considering the regions of maximum response and their respective maximum points in the loadings graph seen in Fig. 3.

**Table 2:** Main selected wavenumbers based on the PCA loadings on PC1, used to distinguish osteosarcopenia samples from healthy controls.

Wavenumber (cm^−1^)	Tentative assignment
1711	C=O
1661	Amide I band
1574	C=N adenine
1510	In-plane CH bending vibration from the phenyl rings; CH in plane bending; Amide II
1398	CH_3_ symmetric deformation
1273	CH $\alpha$ rocking
1225	Collagen; Asymmetric stretching of phosphate groups of phosphodiester linkages in DNA and RNA
1107	CO, CC stretching, ring polysaccharides
906.5	Left-handed helix DNA (Z form)

## Discussion

The use of spectroscopy in the detection and screening of diseases with complex diagnosis has become common in recent years, with excellent results being achieved when combined with multivariate data analysis. For example, the application for the differentiation of breast cancer patients^21^, gestational diabetes mellitus^22^, and even in cases related to physical therapy, such as for the detection of fibromyalgia^23^, with satisfactory accuracy values. A genetic algorithm with linear discriminant analysis model, used to differentiate control patients from those diagnosed with fibomyalgia, obtained an accuracy of 84.2%, with a sensitivity of 89.5% and a specificity of 79%^23^. An SPA-SVM model used for the detection of breast cancer obtained an accuracy of approximately 93%.^21^ In this application to differentiate patients with osteosarcopenia, the model used is a combination of a supervised classifier (SVM) with a dimensionality reduction model (PCA), capable of transforming a large amount of wavenumbers into a few factors built through the linear combination between the original variables, facilitating the interpretation of the results and their visualization through the use of the PCs. The supervised SVM model is quite useful and effective, being used in many cases of difficult separation between classes, presenting satisfactory results in several examples of biospectroscopic applications, such as for classification between patients with prostate cancer^24^ and breast cancer^21^ based on the mid-infrared (MIR) spectral region.

The main wavenumbers responsible for classification are shown in Table 2. Slightly higher absorbance intensities are observed for healthy controls spectra at 1660 cm^−1^, between 1580 to 1400 cm^−1^, and around 1100 cm^−1^ (Fig. 1). These are associated with Amide I of proteins (1661 cm^−1^), C = N of adenine (1574 cm^−1^), Amide II of proteins (1510 cm^−1^), and ring polysaccharides (1107 cm^−1^) vibrations (Table 2). Higher absorbance intensities are observed for the osteosarcopenia group between 1400–1200 cm^−1^ and below 1000 cm^−1^, which are associated with CH_3_ symmetric deformation (1398 cm^−1^), CH $\alpha$ rocking (1273 cm^−1^), collagen and asymmetric stretching of phosphate groups of phosphodiester linkages in DNA and RNA (1225 cm^−1^), and amino acids related to the left-handed helix DNA in Z form (906.5 cm^−1^).

There are several factors associated with osteosarcopenia, including nutrition, lifestyle and genetics, however there are many biochemical changes in the bone-muscle crosstalk that contributes to the development of osteosarcopenia^25^. This includes growth hormone/insulin-like growth factor-1 (GH/IGF-1), gonadal sex hormones and vitamin D, with age-related decreasing contributing to the development of osteosarcopenia^25,26^. Patients with osteosarcopenia have insufficient intake of proteins^25,27^, where reduced levels of protein intake, vitamin D, calcium and reduction in physical activity are correlated with declining muscle strength, thus being key factors for osteosarcopenia^27^. Among these proteins, myokines are small proteins (5–20 kDa) which are fundamental for osteosarcopenia pathogenesis, where altered levels of these proteins lead to disturbance in the balance between anabolic and catabolic effects with consequent age-related muscle atrophy^11^. In addition, genetic polymorphisms of various genes, such as androgen receptor, oestrogen receptor, catechol-O-methyltransferase, IGF-1, vitamin D receptor and low-density-lipoprotein receptor-related protein, contribute to the pathogenesis of osteosarcopenia^25^.

The IR spectra can contain such biohcemical contributions in a complex matrix for which the use of multivariate analysis enable the distinction of case and control groups. For example, changes in protein absorptions and amino acids, such Amide I, Amide II and DNA/RNA absorptions, may be directly associated with the reduction of protein levels and genetic alterations in patients with osteosarcopenia. However, deeper studies are necessary to understand the biochemical pathways of this disease, which may include chromatographic and mass spectrometric techniques, since the FTIR spectra can only provide clues about the functional groups associated with the disease appearance, and do not provide sufficient information to identify specific metabolites or molecular markers associated with the disease. In addition, whilst the results reported herein are promising, ideally such study should be expanded and tested against a larger population of patients to ensure the method can be applied more generally.

## Conclusion

In this study, we were able to distinguish patients with osteosarcopenia from healthy controls based on their blood serum. PCA-SVM results reached 89% accuracy, sensitivity and specificity to distinguish both groups in an external sample test set compared to the gold-standard method. The results are promising and demonstrate the potential of spectroscopic techniques in conjunction with multivariate data analysis for osteosarcopenia diagnosis.
